# Major adverse kidney events in children requiring continuous kidney replacement therapy: a single-center retrospective study in Japan

**DOI:** 10.3389/fped.2026.1757939

**Published:** 2026-02-25

**Authors:** Yusuke Tokuda, Kentaro Ide, Junichiro Morota, Eisaku Nashiki, Kentaro Nishi, Mai Miyaji, Shotaro Matsumoto

**Affiliations:** 1 Critical Care Medicine, National Center for Child Health and Development, Tokyo, Japan; 2 Division of Nephrology and Rheumatology, National Center for Child Health and Development, Tokyo, Japan; 3 Pediatric Intensive Care Unit, Department of Pediatrics, School of Medicine, St. Marianna University, Kanagawa, Japan

**Keywords:** acute kidney injury, continuous kidney replacement therapy, major adverse kidney events, pediatric intensive care unit, pediatrics

## Abstract

**Background:**

Continuous kidney replacement therapy (CKRT) is an essential supportive therapy for children with acute kidney injury. Nevertheless, a considerable proportion of patients fail to recover kidney function and present with major adverse kidney events (MAKE), a composite outcome including death, dialysis dependence, or persistent kidney dysfunction. Recent international pediatric collaborative studies, mainly from North America, have reported on MAKE following pediatric CKRT. However, such data from Japan remain limited.

**Methods:**

We conducted a single-center retrospective study of patients under 16 years of age who received CKRT for renal indications in a tertiary pediatric center between July 2014 and June 2023. The primary outcome was MAKE at 90 days after CKRT initiation (MAKE-90). We used univariate logistic regression analysis to evaluate the association between MAKE-90 and clinical characteristics.

**Results:**

Of the 51 eligible patients, 28 (55%) experienced MAKE-90. The components of MAKE-90 were death in 14 patients (27%), dialysis dependence in 10 (20%), and persistent kidney dysfunction in 4 (8%). Univariate logistic regression analysis revealed that CKRT duration (days) [odds ratio (OR) 1.06, 95% confidence interval (CI) 1.00–1.12] and urine output (mL/kg/h) at 14 days after CKRT initiation (OR 0.33, 95% CI 0.14–0.80) were significantly associated with MAKE-90.

**Conclusion:**

The incidence of MAKE-90 in our Japanese cohort was over half of the children requiring CKRT, comparable to rates reported in international multicenter studies. Longer CKRT duration and lower urine output on day 14 were associated with MAKE-90, suggesting that these factors may serve as potential prognostic markers.

## Introduction

Continuous kidney replacement therapy (CKRT) is a critical supportive therapy in the pediatric intensive care unit (PICU) for children with severe acute kidney injury (AKI). The number of patients requiring CKRT for AKI is increasing worldwide (1). Following an episode of AKI, many patients fail to achieve renal recovery, leading to persistent kidney dysfunction, dialysis dependence, or mortality (2).

Historically, outcomes assessment after kidney replacement therapy focused on single endpoints such as mortality. However, this approach has limitations because death precludes the evaluation of subsequent kidney outcomes. To address this, Major Adverse Kidney Events (MAKE) was proposed as a composite outcome measure that includes death, dialysis dependence, and persistent kidney dysfunction (3). The 90-day time point (MAKE-90) is particularly relevant as it aligns with the timeframe for diagnosing chronic kidney disease and serves as a key indicator of mid-term to long-term renal prognosis.

A recent international pediatric collaborative, the Worldwide Exploration of Renal Replacement Outcomes Collaborative in Kidney Disease (WE-ROCK), reported a MAKE-90 frequency of 65% among 969 children and young adults. This composite was composed of 38% mortality, 9% dialysis dependence, and 27% persistent kidney dysfunction (4). Furthermore, the study identified cardiac comorbidities, delayed CKRT initiation, specific CKRT liberation patterns, and the Pediatric Logistic Organ Dysfunction (PELOD)-2 score at CKRT initiation as independent risk factors (4–6).

Although these international collaborations have provided important insights into MAKE-90 after pediatric CKRT, data from Japan remain limited because pediatric CKRT case volumes are relatively small at individual institutions and are dispersed across centers, which may have contributed to the scarcity of published outcome data from Japan. Given potential differences in healthcare systems, dialysis practices, and patient populations, reporting data from a Japanese cohort is essential. Therefore, this study aimed to describe the frequency of MAKE-90 and identify its associated clinical characteristics in a single Japanese center.

## Methods

### Study design and patients

This single-center, retrospective study was conducted at the National Center for Child Health and Development in Tokyo, Japan. We included pediatric patients under 16 years of age who underwent CKRT for AKI in the PICU between July 2014 and June 2023. We selected patients under 16 years of age to focus on a pediatric PICU population and to obtain results specific to children.

Patients were excluded if they: 1) were receiving chronic maintenance dialysis prior to admission; 2) had severe congenital anomalies of the kidney and urinary tract (CAKUT) resulting in end-stage kidney disease; 3) received CKRT for non-renal indications (e.g., liver failure, inborn errors of metabolism); 4) initiated dialysis at an outside hospital; 5) required concurrent extracorporeal membrane oxygenation (ECMO); or 6) had an unknown 90-day outcome. AKI was diagnosed based on the Kidney Disease Improving Global Outcomes (KDIGO) criteria, with neonatal-specific modifications where appropriate (7, 8). CKRT was initiated when patients developed life-threatening abnormalities that do not respond to medical therapy, including anuria, refractory volume overload, hyperkalemia, high blood urea nitrogen, and refractory metabolic acidosis. In our PICU, CKRT was prescribed using a standardized regimen. The blood flow rate was set at 3–5 mL/kg/min. The dialysate flow rate was set at 25–50 mL/kg/h. Additional hemofiltration was added as needed based on clinical indication. A cellulose triacetate hemofilter was used in all patients. Filter surface area was selected according to body weight and ranged from 0.3 to 2.1 m^2^. For anticoagulation, nafamostat mesylate was used as the first-line agent. Unfractionated heparin was used when clinically indicated. The clinical decision to discontinue dialysis was made by the attending intensivist, based on the patient's clinical status.

### Study variables

We retrospectively collected data from electronic medical records. The variables were selected based on the protocol of a prospective, multicenter CKRT registry in Japanese PICUs (9). Baseline characteristics included sex, age, height, weight, comorbidities, the pediatric index of mortality 2 (PIM2) score, PELOD-2 score at PICU admission, catecholamine use, and the vasoactive-inotropic score (VIS) at CKRT initiation. Clinical and laboratory data at the time of CKRT initiation were also extracted. Given a prior report that urine output trajectories through day 14 are associated with dialysis dependence (10), we collected urine output data at CKRT initiation and on days 3, 7, and 14. Urine output was calculated as a 24-hour average and expressed in mL/kg/h. Data on diuretic administration, fluid balance, and AKI etiology were not available in our database and were not included in the analyses.

### Statistical analysis

The primary outcome was MAKE-90, a composite of the following: 1) death from any cause; 2) dialysis dependence (need for any form of kidney replacement therapy); or 3) persistent kidney dysfunction [a ≥25% decrease in estimated glomerular filtration rate (eGFR) from baseline] at 90 days. If a baseline eGFR was unavailable, a value of 100 mL/min/1.73 m^2^ was imputed, consistent with previous studies (4). Baseline kidney function was often undocumented, particularly in patients without relevant medical history.

Categorical variables are presented as numbers (percentages) and continuous variables as medians [interquartile ranges (IQR)]. We compared clinical characteristics between patients with and without MAKE-90 using Fisher's exact test for categorical variables and the Mann–Whitney U test for continuous variables. A univariate logistic regression analysis was performed to examine the association between clinical characteristics and MAKE-90. Given the limited sample size and event count, we restricted regression analyses to univariate models. All statistical analyses were performed using R software version 4.3.1. Statistical significance was defined as a two-sided *p*-value of less than 0.05.

### Ethics

This study was approved by the Institutional Review Board of the National Center for Child Health and Development (No. 2023-103). In accordance with Japanese ethical guidelines, an opt-out method was used for patient consent. This manuscript was prepared following the Strengthening the Reporting of Observational Studies in Epidemiology (STROBE) guidelines.

## Results

**Figure 1:**
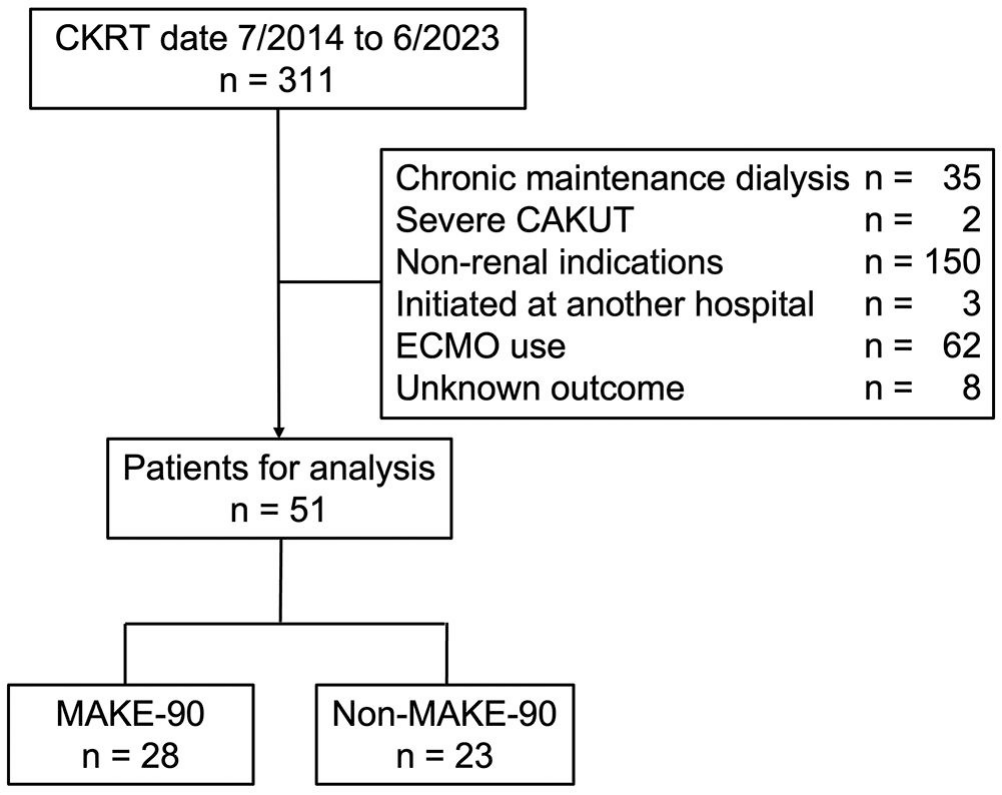
Patient flowchart. CKRT, continuous kidney replacement therapy; CAKUT, congenital anomalies of the kidney and urinary tract; ECMO, extracorporeal membrane oxygenation; MAKE, major adverse kidney events.

During the study period, 311 patients received CKRT, of whom 51 met the inclusion criteria for the final analysis (Figure 1). Of these, 28 patients (55%) experienced MAKE-90 (MAKE-90 group), whereas 23 patients (45%) did not (Non-MAKE-90 group). MAKE-90 components included death in 14 patients (27%), dialysis dependence in 10 (20%), and persistent kidney dysfunction in 4 (8%).

Patient characteristics at CKRT initiation are shown in Table 1. No significant differences were observed in age, sex, or comorbidities between the two groups. Although not statistically significant, patients in the MAKE-90 group tended to have a higher PELOD-2 score at CKRT initiation (median 8.5 vs. 7, *P* = 0.17), a higher frequency of cardiac comorbidities (14% vs. 9%, *P* = 0.68), and nephrological comorbidities (25% vs. 13%, *P* = 0.48).

**Table 1:** Patient characteristics, vital and hematology data at CKRT initiation.

Variable	MAKE-90	Non-MAKE-90	*P* value
	(*n* = 28)	(*n* = 23)	
Male sex	16 (57)	12 (52)	0.78
Age, months	38 (2–100)	61 (17–90)	0.66
Height, cm	94 (57–122)	102 (75–120)	0.43
Body weight, kg	12.2 (5.6–20.9)	18.2 (8.6–22.5)	0.23
Comorbidities			
Cardiac	4 (14)	2 (9)	0.68
Nephrological	7 (25)	3 (13)	0.48
Severity assessment			
PIM2, score	2.0 (0.2–4.5)	1.8 (0.9–6.0)	0.68
PELOD-2, score	8.5 (6–12)	7 (5–10)	0.17
Catecholamine use	13 (46)	7 (30)	0.27
VIS, score	0 (0–15.3)	0 (0–1.5)	0.12
Vital signs at CKRT initiation			
Pulse rate, bpm	120 (107–144)	108 (93–135)	0.16
Systolic blood pressure, mmHg	92 (72–109)	101 (90–113)	0.18
Diastolic blood pressure, mmHg	50 (43–64)	53 (48–66)	0.34
Urine output, mL/kg/h	0.3 (0.1–1.2)	0.3 (0.2–0.5)	0.91
Hematology data at CKRT initiation			
pH	7.29 (7.24–7.37)	7.36 (7.32–7.39)	0.08
Lactate, mmol/L	1.9 (0.8–5.6)	1.2 (0.8–3.5)	0.51
Bicarbonate, mmol/L	21.1 (17.2–22.8)	21.1 (17.5–23.3)	0.73
Base excess, mmol/L	–5.7 (–7.9––2.7)	–4.3 (–7.7––0.1)	0.47
Sodium, mEq/L	138 (134–145)	138 (133–144)	0.93
Potassium, mEq/L	4.3 (3.7–5.3)	3.8 (3.3–4.6)	0.22
Chloride, mEq/L	106 (101–112)	105 (99–108)	0.48
Hemoglobin, g/dL	9.3 (8.2–12.3)	9.7 (8.6–11.3)	0.96
Platelet, 10^4^/µL	8.9 (3.8–25.4)	8.3 (5.6–21.5)	0.97
Creatinine, mg/dL	1.7 (0.9–3.2)	1.6 (0.7–5.4)	0.60
Blood urea nitrogen, mg/dL	46.2 (21.2–64.2)	66.7 (36.5–87.5)	0.10

Data are presented as *n* (%) or median (interquartile range).

CKRT, continuous kidney replacement therapy; MAKE, major adverse kidney events; PELOD, pediatric logistic organ dysfunction; PIM, pediatric index of mortality; VIS, vasoactive-inotropic score.

There were no significant differences in the time from ICU admission to CKRT initiation (2 days vs. 2 days, *P* = 0.92) or the length of ICU stay (18 days vs. 14 days, *P* = 0.76). However, the duration of dialysis was significantly longer in the MAKE-90 group than in the non-MAKE-90 group (17 days vs. 3 days, *P* = 0.003). Urine output (mL/kg/h) at 3, 7, and 14 days after CKRT initiation was significantly lower in the MAKE-90 group (Day 3: 0.05 vs. 0.33, *P* = 0.009; Day 7: 0.05 vs. 1.27, *P* = 0.002; Day 14: 0.1 vs. 1.6, *P* = 0.006). Urine output data were available for 24/28, 22/28, and 18/28 patients in the MAKE-90 group and for 23/23, 19/23, and 16/23 patients in the non-MAKE-90 group on days 3, 7, and 14, respectively.

The results of the univariate logistic regression analysis are presented in Table 2. CKRT duration (days) [odds ratio (OR) 1.06, 95% confidence interval (CI) 1.00–1.12] and urine output (mL/kg/h) on day 14 (OR 0.33, 95% CI 0.14–0.80) were associated with MAKE-90.

**Table 2:** Logistic regression analysis of factors associated with MAKE-90.

Variable	**Odds ratio**	**95% CI**	***P* value**
Age, months	1.00	0.99–1.01	0.87
Primary comorbidity: cardiac	1.75	0.29–10.5	0.54
PELOD-2 score at CKRT initiation	1.11	0.95–1.31	0.19
Time from ICU admission to CKRT initiation	0.99	0.93–1.05	0.69
CKRT duration	1.06	1.00–1.12	0.03
Urine output, mL/kg/h			
Day 3	0.35	0.12–1.04	0.06
Day 7	0.61	0.35–1.07	0.08
Day 14	0.33	0.14–0.80	0.01

MAKE, major adverse kidney events; PELOD, pediatric logistic organ dysfunction; ICU, intensive care unit; CKRT, continuous kidney replacement therapy.

## Discussion

In this single-center retrospective study, we investigated the frequency and clinical factors associated with MAKE-90 following pediatric CKRT in a Japanese PICU. We found that 55% of patients experienced MAKE-90, and longer CKRT duration and lower urine output on day 14 after CKRT initiation were associated with this adverse outcome.

The overall frequency of MAKE-90 in our cohort was more than half of the children, and comparable to the 65% reported in the multinational WE-ROCK study. While the overall frequency was similar, the composition of MAKE-90 differed between our cohort and the WE-ROCK study (Table 3) (4). Our cohort showed a non-significant trend toward lower mortality (27% vs. 38%, *P* = 0.14), but had a significantly higher rate of dialysis dependence (20% vs. 9%, *P* = 0.03). Several factors may account for these differences. First, our cohort included a higher proportion of patients with nephrological comorbidities compared to the WE-ROCK cohort [20% (10/51) vs. 9% (91/969), *P* = 0.03], which may have contributed to a higher rate of dialysis dependence. Second, differences in healthcare systems may also contribute to these disparities. In Japan, the out-of-pocket cost of dialysis is markedly reduced through comprehensive social security programs (11, 12), which substantially alleviate the financial burden on patients and their families. This supportive system may be associated with a more cautious approach to dialysis liberation, and may partly explain the higher rate of dialysis dependence observed in our cohort.

**Table 3:** Frequency of MAKE-90 compared with the international study.

Outcome	Our cohort	WE-ROCK	*P* value
MAKE-90	28/51 (55%)	630/969 (65%)	0.18
Mortality	14/51 (27%)	368/969 (38%)	0.14
Dialysis dependence	10/51 (20%)	91/969 (9%)	0.03
Persistent kidney dysfunction^a^	4/51 (8%)	171/969 (18%)	0.08

^a^ Persistent kidney dysfunction excludes dialysis dependence.

*P* values were calculated in the present study by comparing our cohort with proportions reported in WE-ROCK.

MAKE, major adverse kidney events; WE-ROCK, Worldwide Exploration of Renal Replacement Outcomes Collaborative in Kidney Disease.

Regarding the clinical factors associated with MAKE-90, logistic regression analysis demonstrated that longer CKRT duration and lower urine output on day 14 were associated with MAKE-90. Regarding CKRT duration, dialysis dependence is a component of the MAKE definition, and CKRT duration is therefore not fully independent of the outcome. However, dialysis-related organ injury has been reported as a potential factor contributing to MAKE, including renal hypoperfusion due to intradialytic hypotension or excessive fluid removal (13–15), as well as hemorrhagic complications from anticoagulation and an increased risk of catheter-related infections (16, 17). Therefore, it should also be considered that a longer duration of CKRT itself may have detrimental effects on renal recovery and overall outcomes. The role of urine output for successful liberation from dialysis is well-established (10, 13). As the diuretic phase of AKI recovery typically occurs within one to two weeks (18), a lack of meaningful urine output by day 14 may indicate impaired renal recovery and a poor long-term prognosis. Although limited statistical power may partly explain the nonsignificant associations on days 3 and 7, urine output at these earlier time points may be less discriminative because some patients may not yet have entered the diuretic phase. Thus, urine output at this time point could serve as a practical and important prognostic marker to help identify high-risk patients and guide prognostic discussions with patients and families.

This study has several limitations. First, its single-center, retrospective design and small sample size limited our ability to perform multivariable analyses to adjust for confounders, and residual confounding is possible. Second, decisions regarding CKRT discontinuation were determined at the discretion of the attending physicians rather than standardized criteria, which may limit the generalizability of our findings. Larger, multicenter studies across Japan are warranted to validate these observations, which could help refine clinical strategies and provide more informed prognostic information for patients and their families. Third, because our database was restricted to patients younger than 16 years according to the pediatric definition in Japan, differences in age range compared with international studies may confound comparisons of outcomes.

## Conclusion

In this single-center Japanese cohort, MAKE-90 occurred in more than half of the children requiring CKRT, a rate comparable to that reported in international studies. Longer CKRT duration and lower urine output 14 days after CKRT initiation were associated with MAKE-90, suggesting that these factors may serve as potential prognostic markers.

## Data Availability

The raw data supporting the conclusions of this article will be made available by the authors, without undue reservation.
